# Changes in Association between Previous Therapeutic Abortion and Preterm Birth in Scotland, 1980 to 2008: A Historical Cohort Study

**DOI:** 10.1371/journal.pmed.1001481

**Published:** 2013-07-09

**Authors:** Clare Oliver-Williams, Michael Fleming, Kirsten Monteath, Angela M. Wood, Gordon C. S. Smith

**Affiliations:** 1 Department of Public Health and Primary Care, University of Cambridge, Cambridge, United Kingdom; 2 Information Services Division, NHS National Services Scotland, Edinburgh, United Kingdom; 3 Department of Obstetrics and Gynaecology, National Institute for Health Research Biomedical Research Centre, University of Cambridge, Cambridge, United Kingdom; WHO, United States of America

## Abstract

**Background:**

Numerous studies have demonstrated that therapeutic termination of pregnancy (abortion) is associated with an increased risk of subsequent preterm birth. However, the literature is inconsistent, and methods of abortion have changed dramatically over the last 30 years. We hypothesized that the association between previous abortion and the risk of preterm first birth changed in Scotland between 1 January 1980 and 31 December 2008.

**Methods and Findings:**

We studied linked Scottish national databases of births and perinatal deaths. We analysed the risk of preterm birth in relation to the number of previous abortions in 732,719 first births (≥24 wk), adjusting for maternal characteristics. The risk (adjusted odds ratio [95% CI]) of preterm birth was modelled using logistic regression, and associations were expressed for a one-unit increase in the number of previous abortions. Previous abortion was associated with an increased risk of preterm birth (1.12 [1.09–1.16]). When analysed by year of delivery, the association was strongest in 1980–1983 (1.32 [1.21–1.43]), progressively declined between 1984 and 1999, and was no longer apparent in 2000–2003 (0.98 [0.91–1.05]) or 2004–2008 (1.02 [0.95–1.09]). A statistical test for interaction between previous abortion and year was highly statistically significant (*p*<0.001). Analysis of data for abortions among nulliparous women in Scotland 1992–2008 demonstrated that the proportion that were surgical without use of cervical pre-treatment decreased from 31% to 0.4%, and that the proportion of medical abortions increased from 18% to 68%.

**Conclusions:**

Previous abortion was a risk factor for spontaneous preterm birth in Scotland in the 1980s and 1990s, but the association progressively weakened and disappeared altogether by 2000. These changes were paralleled by increasing use of medical abortion and cervical pre-treatment prior to surgical abortion. Although it is plausible that the two trends were related, we could not test this directly as the data on the method of prior abortions were not linked to individuals in the cohort. However, we speculate that modernising abortion methods may be an effective long-term strategy to reduce global rates of preterm birth.

*Please see later in the article for the Editors' Summary*

## Abstract 2

Gordon C. Smith and colleagues used national databases to investigate the association between previous termination of pregnancy and preterm birth in Scotland between 1980 to 2008, and whether the type of procedure was an important factor.

*Please see later in the article for the Editors' Summary*

## Editors' Summary

**Background:**

Therapeutic termination of pregnancy is relatively common, with an estimated 40 million procedures performed worldwide every year. Until two decades ago, most terminations were performed as a surgical procedure, but now the majority of terminations are medically induced with a combination of drugs—selective progesterone receptor antagonists, such as mifepristone, and prostaglandins—that cause less damage to the woman's cervix. Although surgical terminations are still performed, nowadays prostaglandins are also used to help prevent damage to the cervix. Protecting the woman's cervix can help to reduce the risk of spontaneous preterm birth (delivery before 37 weeks gestation) in subsequent pregnancies. As many women who have abortions go on to have subsequent births, the widespread use of termination may be a significant factor in the high global rates of preterm delivery.

**Why Was This Study Done?:**

A previous meta-analysis (a study that combines information from several studies) showed that the risk of preterm delivery was higher in women who had had a previous termination compared to women who had not. Based on this meta-analysis, UK guidelines on the care of women requesting a termination currently recommend that they be informed of the increased risk of subsequent preterm birth. However, it is biologically plausible that women undergoing medical termination or current practice for surgical termination (using prostaglandins to protect and prepare the cervix) may not have an increased risk of subsequent preterm delivery, because such approaches may cause less trauma to the cervix than traditional surgical termination. So in this study, the researchers used a large dataset from Scotland with three decades of information about previous terminations and subsequent preterm deliveries to test this possibility.

**What Did the Researchers Do and Find?:**

The researchers linked two national databases—the Scottish Morbidity Record 02 (SMR02), which records the clinical and demographic characteristics and outcomes of all patients giving birth in Scottish maternity hospitals, and the Scottish Stillbirth and Infant Death Survey (SSBIDS), which classifies all perinatal deaths in Scotland. SMR02 data were available from 1980 onwards and also recorded each woman's self-reported total number of previous abortions, and SSBIDS data were available from 1985. Then the researchers used information from NHS National Services Scotland to examine secular trends in terminations over the past few decades, specifically, whether a recorded termination was surgical or medical, and also whether surgical abortion was preceded by cervical preparation.

Using these methods, the researchers identified that there were 757,060 live, singleton first births between 1980 and 2008 and that 56,816 women reported one previous termination, 5,790 women reported two previous terminations, and 822 women reported three previous terminations. In their analysis (adjusted for maternal characteristics) the researchers found that there was an independent association of spontaneous preterm birth, but not induced preterm birth, with previous termination. The researchers calculated that the chance (odds) of spontaneous preterm birth for one, two, and three or more previous abortions was 1.17, 1.51, and 1.64, after adjusting for maternal characteristics, including smoking. Over the time period, the researchers found that the proportion of surgical terminations without use of cervical pre-treatment decreased from 31% in 1992 to 0.4% in 2008, and over the same period the proportion of medical terminations increased from 18% to 68%. These trends are important, because in their analysis by year of delivery, the researchers found that the association between preterm delivery and previous termination was strongest in 1980–1983, progressively declined between 1984 and 1999, and was no longer present from 2000 to 2008.

**What Do These Findings Mean?:**

These findings support the established association between previous termination and preterm delivery. But most importantly, the changes in this association over the past two decades—from strong in 1980–1983 to nonexistent in 2000–2008—a period in which the use of medical termination and pre-treatment of the cervix for surgical termination increased dramatically in Scotland, suggest that surgical termination without cervical pre-treatment is responsible for the increased risk of spontaneous preterm birth: the decrease in the proportion of this procedure over the study period may have led to the disappearance of the established association between previous termination and preterm delivery from 2000 onwards. However, these findings are limited in that the researchers could not directly test whether the two trends were related because they did not have information on the method of previous termination linked to subsequent birth outcome for individual women. However, based on the findings of this study, it is possible that using modern methods of termination of pregnancy (rather than purely surgical methods) could be a factor in reducing global rates of spontaneous preterm delivery in the future.

**Additional Information:**

Please access these websites via the online version of this summary at http://dx.doi.org/10.1371/journal.pmed.1001481.

- Wikipedia gives more information about termination of pregnancy (note that Wikipedia is a free online encyclopedia that anyone can edit; available in several languages)
- More information is available about the SMR02 dataset used in this study
- The World Health Organization gives information on preterm birth

## Introduction

Therapeutic termination of pregnancy (abortion) remains relatively common, with over 40 million procedures performed worldwide [1]. Historically, abortions were performed as surgical procedures and involved purely mechanical dilation of the cervix, which could lead to cervical injury. As cervical function is key to successful pregnancy, it is plausible that purely surgical abortion could be causally related to the risk of spontaneous premature birth in subsequent ongoing pregnancies. A meta-analysis in 2009 concluded that, compared with women with no previous abortion, the risk (adjusted odds ratio [95% CI]) of spontaneous preterm birth was increased among women with a history of one previous induced abortion (1.27 [1.12–1.44]) and was greater still among women with two or more previous abortions (1.62 [1.27–2.07]) [2]. Given that many women who have abortions go on to have subsequent births, it is likely that the widespread use of abortion is a significant determinant of global rates of preterm delivery. Current guidelines on the care of women requesting abortion recommend that they should be informed of the increased risk of subsequent preterm birth [3].

Developments in the understanding of the biology of parturition led to important modifications in methods of abortion. Understanding the role of endogenous prostaglandins in the control of the cervix during parturition led to the use of analogues of E series prostaglandins (e.g., gemeprost and misoprostol) to soften and dilate the cervix prior to surgical abortion [4]. Moreover, the recognition of the key role of progesterone in maintaining pregnancy led to the development of selective progesterone receptor antagonists (e.g., mifepristone), often used in combination with prostaglandins, for medical abortion [5]. Both approaches would be presumed to be less likely to lead to cervical trauma, and, hence, it is plausible that both might ameliorate the risk of subsequent preterm birth. We hypothesized that increased use of these methods of abortion in a population would reduce or eliminate the association between previous abortion and the risk of preterm birth. We studied nationally collected data from Scotland from 1980 to 2008 to test this hypothesis.

## Methods

### Study Populations

We studied the linked Scottish Morbidity Record 02 (SMR02) and Scottish Stillbirth and Infant Death Survey (SSBIDS). The SMR02 records the clinical and demographic characteristics and outcomes of all patients giving birth in Scottish maternity hospitals, and the SSBIDS classifies all perinatal deaths in Scotland. Approval for the record linkage was provided by the Privacy Advisory Committee of the Information Services Division of the National Health Service (NHS) National Services Scotland. SMR02 data were available from 1980 onwards, and SSBIDS data were available from 1985 onwards. We studied first births that occurred at or after 24 wk gestation. Each woman's total prior number of abortions was ascertained by self report at the first antenatal visit and was recorded in the SMR02.

Secular trends in the methods used for abortions were obtained from the Information Services Division of NHS National Services Scotland, which collects national statistics, separate from the maternity database, on the methods of abortion, through statutory notifications required under the Abortion (Scotland) Regulations 1991. These data were not linked to the SMR02 in the present study, but were simply used to determine temporal changes in the procedures employed. The notifications included information on the method of abortion, which was >99% complete from 1992 onwards. Specifically, it documented whether an abortion was surgical or medical, and whether surgical abortion was preceded by “cervical preparation”. The exact method of cervical preparation was not documented, but synthetic prostaglandins were the conventional method during this time period. Surgical abortion was defined as vacuum aspiration, dilatation and curettage, and others. Medical abortion was defined as use of prostaglandins, oxytocics, and/or anti-progesterones (mifepristone was licensed for use in Scotland in July 1991). Cases of attempted medical abortion that ultimately required surgical evacuation of the uterus were defined as medical procedures.

### Definitions

All maternal characteristics were defined on the basis of the SMR02 record. Preterm birth was defined as birth before 37 wk gestation and was also subdivided into spontaneous and induced preterm delivery. Induced preterm births were defined as those where there was either a pre-labour caesarean section or a documented method of induction of labour. Socioeconomic deprivation was measured using the Carstairs socioeconomic deprivation score, a scoring system based on census data on car ownership, unemployment, overcrowding, and social class within postcode sectors of residence, which contain approximately 1,600 residents [6]. These scores were then classified into seven categories (1 = least deprived, 7 = most deprived). Calendar year of delivery (1 January to 31 December) was segregated into 4-y epochs, with the exception of the final category, which included 5 y. Height, measured in centimetres, was evaluated at the first antenatal visit. Smoking during pregnancy, history of miscarriage, and marital status were self-reported at the first antenatal visit. Smoking was defined as current, never, or ex-smoker. Miscarriage was defined as a pregnancy that ended spontaneously with the loss of a non-registerable fetus (defined as less than 28 wk until 30 September 1992 and less than 24 wk thereafter). Marital status was defined as married or other (co-habiting, divorced, widowed, or single). Maternal age was defined as the mother's age on the day of her child's birth. All perinatal death data were defined on the basis of the SSBIDS record. Neonatal death was defined as death of a live-born infant in the first 28 d of life. Neonatal deaths due to congenital abnormality or Rhesus disease were excluded.

### Statistical Analyses

Continuous variables were compared using the Kruskal-Wallis test, and categorical variables were compared using the χ^2^ test. The risks of preterm birth and neonatal death were analysed using logistic regression. Interactions were assessed using the Wald test. The main interaction of interest was between number of previous abortions and the year of delivery. However, we also assessed the specificity of any change in association over the time period by studying an interaction between height and year of delivery. We have previously shown that short stature is a risk factor for spontaneous preterm birth in Scotland [7], and this has also been confirmed in a meta-analysis of more than 50 studies from around the world [8]. When height was used as a primary exposure, odds ratios were reported for a 10-cm decrease in maternal height.

In order to demonstrate the consistency of associations, history of previous abortion was treated as a continuous variable (truncated at three previous abortions because of the small numbers of women with more than three previous procedures), as a binary variable (any previous abortions versus no previous abortions), and as a categorical variable. However, the main focus of analysis involved its treatment as a continuous variable. Year of birth was treated continuously when assessing the interaction between previous abortion and year of delivery. However, in order to illustrate the interaction graphically, odds ratios for previous abortion were stratified by epoch of delivery.

Where the proportion of cases with missing values for a given covariate was small, records with missing data were excluded because they would have had a minimal effect on the results of the analysis but would have significantly increased its complexity. Where a significant proportion of values was missing, missing data were imputed using multiple imputation by chained equations (used for height, marital status, and smoking) [9]. Five imputations were created using a set of appropriate imputation models constructed of all covariates and outcome variables, stratified by epoch. An additional 35 imputations were created to evaluate whether the results were robust to changes in the number of imputations used. Cox regression was used to determine whether associations between previous abortion and preterm birth varied across the gestational age range 24 to 36 wk, as previously described [7]. Gestational age was used as the time scale, spontaneous preterm labour was the event, induced preterm labour was treated as censored, and all deliveries beyond 36 wk were censored at 37 wk. The proportional hazards assumption was assessed using the test of Grambsch and Therneau [10]. *p*-Values for all hypothesis tests were two-sided, and statistical significance was set at *p*<0.05. All statistical analyses were performed using the Stata software package, version 12.1 (Stata Corporation).

## Results

**Figure 1:**
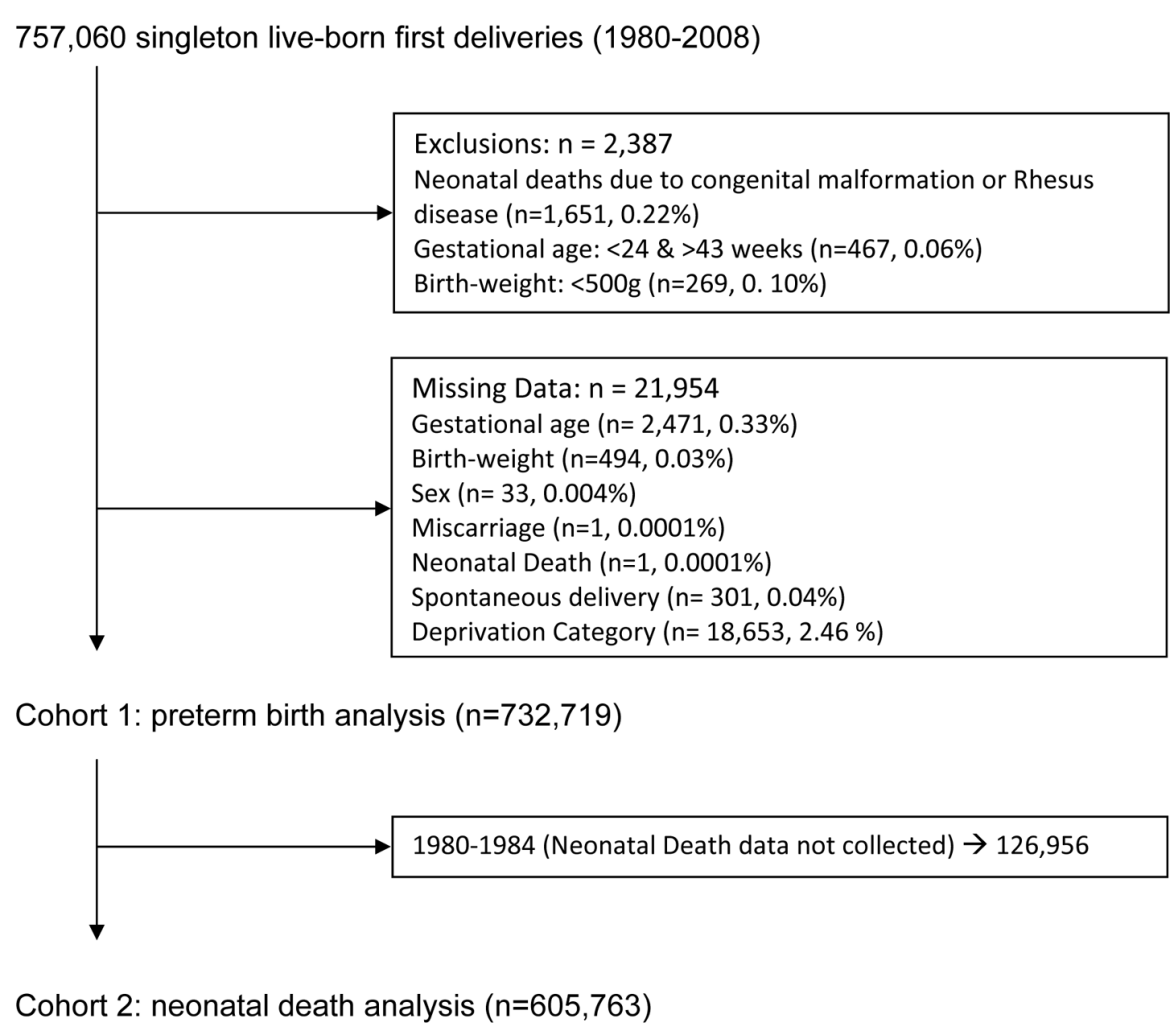
Selection of the study cohorts.

The linked databases identified 757,060 records of singleton, live first births between 1 January 1980 and 31 December 2008. A study cohort of 732,719 records was selected following application of inclusion and exclusion criteria (Figure 1). As neonatal death data from the SSBIDS were available only from 1985, a sub-group of 605,763 births from 1985 to 2008 was also selected. The maternal, obstetric, and infant characteristics of the cohort are tabulated by the number of previous abortions in Table 1. All characteristics varied by the number of previous abortions, as did the proportion of preterm births and neonatal deaths associated with preterm delivery.

**Table 1:** Maternal characteristics and outcomes in relation to the number of previous abortions.

Characteristic or Outcome	All (*n* = 732,719)		Number of Previous Abortions								*p*-Value
			0 (*n* = 669,291)		1 (*n* = 56,816)		2 (*n* = 5,790)		≥3 (*n* = 822)		
**Characteristics**											
**Median height** a **in centimetres (IQR)**	162	(157–167)	162	(157–167)	162	(158–167)	163	(158–167)	163	(160–168)	<0.001
**Median age in years (IQR)**	25	(21–29)	25	(21–29)	26	(22–30)	27	(24–32)	29	(25–33)	<0.001
**Median gestation in weeks (IQR)**	40	(39–41)	40	(39–41)	40	(39–41)	40	(39–41)	40	(38–41)	<0.001
**Smoking status** a ^,^ b											
Never	234,334	(58.0)	215,955	(58.9)	16,439	(50.1)	1,711	(45.2)	229	(39.7)	<0.001
Current	122,520	(30.3)	109,383	(29.8)	11,447	(34.9)	1,449	(38.3)	241	(41.8)	
Ex-smoker	46,995	(11.6)	41,306	(11.3)	4,957	(15.1)	625	(16.5)	107	(18.5)	
**Socioeconomic deprivation category**											
1 (least deprived)	36,552	(5.0)	33,311	(5.0)	2,851	(5.0)	334	(5.8)	56	(6.8)	<0.001
2	98,408	(13.4)	88,703	(13.3)	8,561	(15.1)	984	(17.0)	160	(19.5)	
3	148,507	(20.3)	135,283	(20.2)	11,818	(20.8)	1,230	(21.2)	176	(21.4)	
4	183,078	(25.0)	166,731	(24.9)	14,665	(25.8)	1,477	(25.5)	205	(24.9)	
5	116,746	(15.9)	107,043	(16.0)	8,732	(15.4)	857	(14.8)	114	(13.9)	
6	89,449	(12.2)	82,272	(12.3)	6,506	(11.5)	596	(10.3)	75	(9.1)	
7 (most deprived)	59,979	(8.2)	55,948	(8.4)	3,683	(6.5)	312	(5.4)	36	(4.4)	
**Marital status** a											
Married	407,370	(61.5)	376,036	(62.3)	28,555	(54.5)	2,476	(47.4)	303	(41.5)	<0.001
Other	254,764	(38.5)	227,781	(37.7)	23,813	(45.5)	2,743	(52.6)	427	(58.5)	
**Miscarriage**											
None	646,382	(88.2)	593,108	(88.6)	48,051	(84.6)	4,597	(79.4)	626	(76.2)	<0.001
History of miscarriage	86,337	(11.8)	76,183	(11.4)	8,765	(15.4)	1,193	(20.6)	196	(23.8)	
**Epoch**											
1980–1983	101,672	(13.9)	95,278	(14.2)	6,003	(10.6)	364	(6.3)	27	(3.3)	<0.001
1984–1987	103,693	(14.2)	95,236	(14.2)	7,798	(13.7)	582	(10.1)	77	(9.4)	
1988–1991	112,981	(15.4)	102,242	(15.3)	9,796	(17.2)	851	(14.7)	92	(11.2)	
1992–1995	106,212	(14.5)	96,059	(14.4)	9,078	(16.0)	964	(16.6)	111	(13.5)	
1996–1999	97,970	(13.4)	88,318	(13.2)	8,524	(15.0)	983	(17.0)	145	(17.6)	
2000–2003	88,768	(12.1)	80,142	(12.0)	7,536	(13.3)	932	(16.1)	158	(19.2)	
2004–2008	121,423	(16.6)	112,016	(16.7)	8,081	(14.2)	1,114	(19.2)	212	(25.8)	
**Outcomes**											
**Neonatal death**											
All^b^	1,214	(0.2)	1,091	(0.2)	107	(0.2)	12	(0.2)	4	(0.5)	0.05
Preterm^b^	874	(0.1)	790	(0.1)	71	(0.1)	9	(0.2)	4	(0.5)	0.02
Term^b^	340	(<0.1)	301	(<0.1)	36	(0.1)	3	(0.1)	0	(0.0)	0.24
**Preterm birth**											
All	45,967	(6.3)	41,456	(6.2)	3,950	(7.0)	485	(8.4)	76	(9.2)	<0.001
Spontaneous	30,462	(4.2)	27,370	(4.1)	2,699	(4.8)	342	(5.9)	51	(6.2)	<0.001
Induced	15,505	(2.1)	14,086	(2.1)	1,251	(2.2)	143	(2.5)	25	(3.0)	0.01
24–28 wk	2,929	(0.4)	2,631	(0.4)	239	(0.4)	48	(0.8)	11	(1.3)	<0.001
29–32 wk	7,156	(1.0)	6,479	(1.0)	593	(1.0)	72	(1.2)	12	(1.5)	0.01
29–32 wk	35,882	(4.9)	32,346	(4.8)	3,118	(5.5)	365	(6.3)	53	(6.4)	0.01

Data are *n* (percent) unless otherwise indicated.

a Number (percent) missing data: height, 106,661 (14.6); marital status, 70,585 (9.6); smoking status (1992–2008), 43,998 (10.6).

b Sub-group of births was used to calculate proportions: neonatal death data were available from 1985 onwards, and smoking data were available from 1992 onwards.

IQR, inter-quartile range.

**Figure 2:**
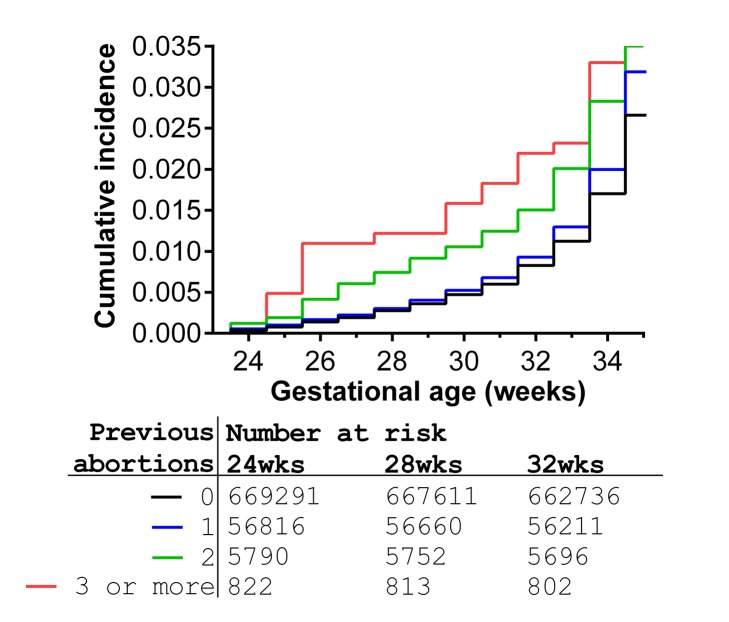
Cumulative incidence of preterm birth from 24 wk onwards in relation to number of previous abortions for 732,719 nulliparous women, Scotland 1980–2008. The relative risk of preterm birth for women with zero, one, two, or three or more previous abortions significantly varied across the range 24 to 36 wk gestational age (global test of proportional hazards assumption: *p* = 0.02). The graph is confined to the risk prior to 34 wk to allow better visualisation of the differences in incidence of extreme preterm births.

There was a positive association between preterm birth and previous therapeutic abortion in the univariate analysis (Table 2). In univariate analysis, there was a 15% increase in the odds of preterm birth associated with each previous abortion. When adjusted for maternal characteristics, there was an 18% increase in the odds of spontaneous preterm birth for each prior abortion, but no association between previous abortion and the risk of induced preterm birth. The strength of the association between prior abortion and the risk of spontaneous preterm birth was compared across the range 24 to 36 wk (Figure 2). A Cox model demonstrated that the hazard ratio for prior abortion significantly varied across this range (test for non-proportionality: *p* = 0.02). The associations for two previous abortions and three or more previous abortions were stronger at 24–28 wk than later gestational ages (Table 2). Moreover, women with three or more previous abortions had an adjusted odds ratio (95% CI) for neonatal death of 2.68 (1.00–7.20). Further adjustment for the week of gestational age at delivery resulted in complete loss of this association (1.01 [0.32–3.16]), indicating that the increased risk of neonatal death was explained by the increased risk of preterm birth in these women. All associations between history of previous abortion treated as a categorical variable, as a binary variable, and as a continuous variable are tabulated along with all the adjusted odds ratios for all maternal covariates in Tables S1, S2, S3, respectively.

**Table 2:** Logistic regression analysis of the association between previous abortion and the risk of preterm birth and neonatal death.

Outcome	Unadjusted Odds Ratios (95% CI)						Adjusted^a^ Odds Ratios (95% CI)					
	History of Abortion	Number of Previous Abortions			Per-Unit Increase^b^	*p*-Value^c^	History of Abortion	Number of Previous Abortions			Per-Unit Increase^b^	*p*-Value^c^
		1	2	≥3				1	2	≥3		
**Preterm birth**												
All	1.16 (1.12–1.20)	1.13 (1.09–1.17)	1.38 (1.26–1.52)	1.54 (1.22–1.95)	1.15 (1.12–1.18)	<0.001	1.13 (1.10–1.17)	1.11 (1.07–1.15)	1.32 (1.20–1.45)	1.45 (1.15–1.84)	1.12 (1.09–1.16)	<0.001
Spontaneous	1.20 (1.16–1.25)	1.17 (1.12–1.22)	1.48 (1.32–1.65)	1.57 (1.18–2.08)	1.18 (1.15–1.22)	<0.001	1.21 (1.16–1.26)	1.17 (1.13–1.22)	1.51 (1.35–1.68)	1.64 (1.24–2.18)	1.19 (1.15–1.23)	<0.001
Induced	1.07 (1.02–1.13)	1.05 (0.99–1.12)	1.20 (1.02–1.42)	1.49 (1.00–2.23)	1.08 (1.03–1.13)	0.002	1.00 (0.95–1.06)	1.00 (0.94–1.06)	1.05 (0.86–1.21)	1.17 (0.78–1.74)	1.01 (0.96–1.05)	0.77
24–28 wk	1.21 (1.07–1.36)	1.08 (0.94–1.23)	2.16 (1.62–2.88)	3.52 (1.94–6.39)	1.27 (1.15–1.39)	<0.001	1.17 (1.04–1.32)	1.05 (0.92–1.20)	2.05 (1.54–2.74)	3.33 (1.83–6.06)	1.24 (1.12–1.36)	<0.001
29–32 wk	1.11 (1.03–1.21)	1.09 (1.00–1.18)	1.32 (1.04–1.66)	1.56 (0.88–2.76)	1.11 (1.04–1.19)	0.002	1.08 (1.00–1.17)	1.06 (0.98–1.16)	1.25 (0.99–1.58)	1.46 (0.82–2.59)	1.09 (1.02–1.16)	0.02
33–36 wk	1.16 (1.12–1.21)	1.14 (1.10–1.19)	1.34 (1.20–1.49)	1.38 (1.04–1.82)	1.14 (1.11–1.18)	<0.001	1.14 (1.10–1.18)	1.13 (1.08–1.17)	1.28 (1.15–1.42)	1.30 (0.98–1.72)	1.12 (1.09–1.16)	<0.001
**Neonatal death**	1.13 (0.94–1.36)	1.10 (0.90–1.34)	1.15 (0.65–2.02)	2.59 (0.97–6.94)	1.13 (0.97–1.32)	0.11	1.10 (0.91–1.33)	1.07 (0.88–1.31)	1.14 (0.64–2.01)	2.68 (1.00–7.20)	1.11 (0.95–1.30)	0.17

a Adjusted for maternal height, age, history of miscarriage, marital status, socioeconomic status, and year of delivery.

b Expressed as 0, 1, 2, and 3 or more.

c *p*-Value for trend.

**Figure 3:**
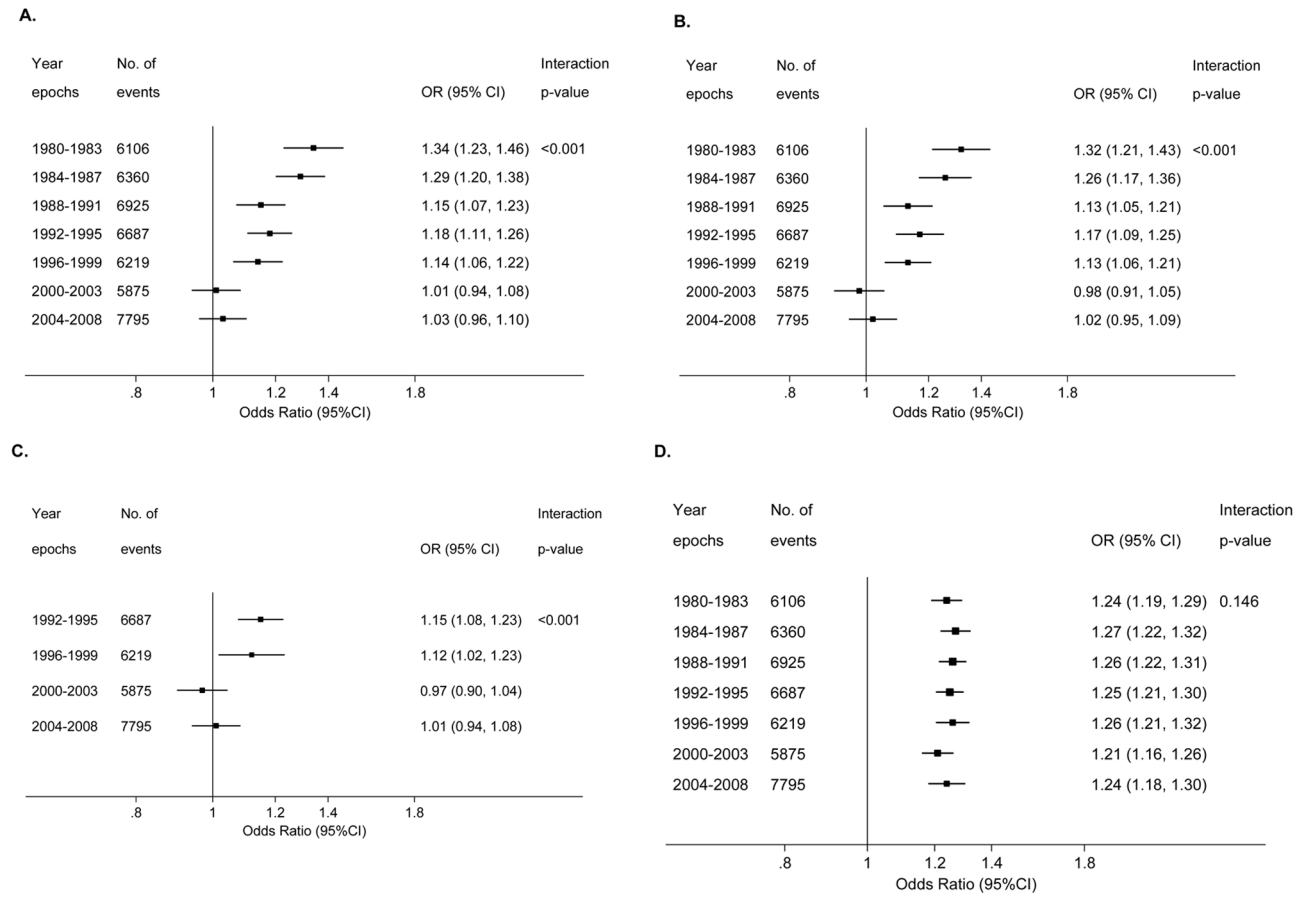
Forest plots of odds ratios for preterm birth in Scotland by epoch. (A) Unadjusted odds ratio for a one-unit increase in number of previous abortions (coded as 0, 1, 2, and 3 or more) in relation to risk of preterm first birth among 732,719 women for births from 1980 to 2008. (B) As in (A), but odds ratio adjusted for maternal characteristics (deprivation category, previous miscarriage, maternal age, height, and marital status). (C) Adjusted odds ratio for a one-unit increase in number of previous abortions in relation to risk of preterm first birth among 414,373 women for births from 1992 to 2008. Odds ratios adjusted for maternal characteristics as in (B), but also for smoking. (D) Adjusted odds ratio for a 10-cm decrease in maternal height in relation to the risk of preterm first birth among 732,719 women for births from 1980 to 2008. Odds ratios adjusted for deprivation category, maternal age, marital status, previous abortion, and previous miscarriage. The interaction *p*-value is for a Wald test of the null hypothesis that the odds ratios did not significantly differ across the period 1980 to 2008. Year is treated as a continuous variable in all the statistical tests of interaction.

**Figure 4:**
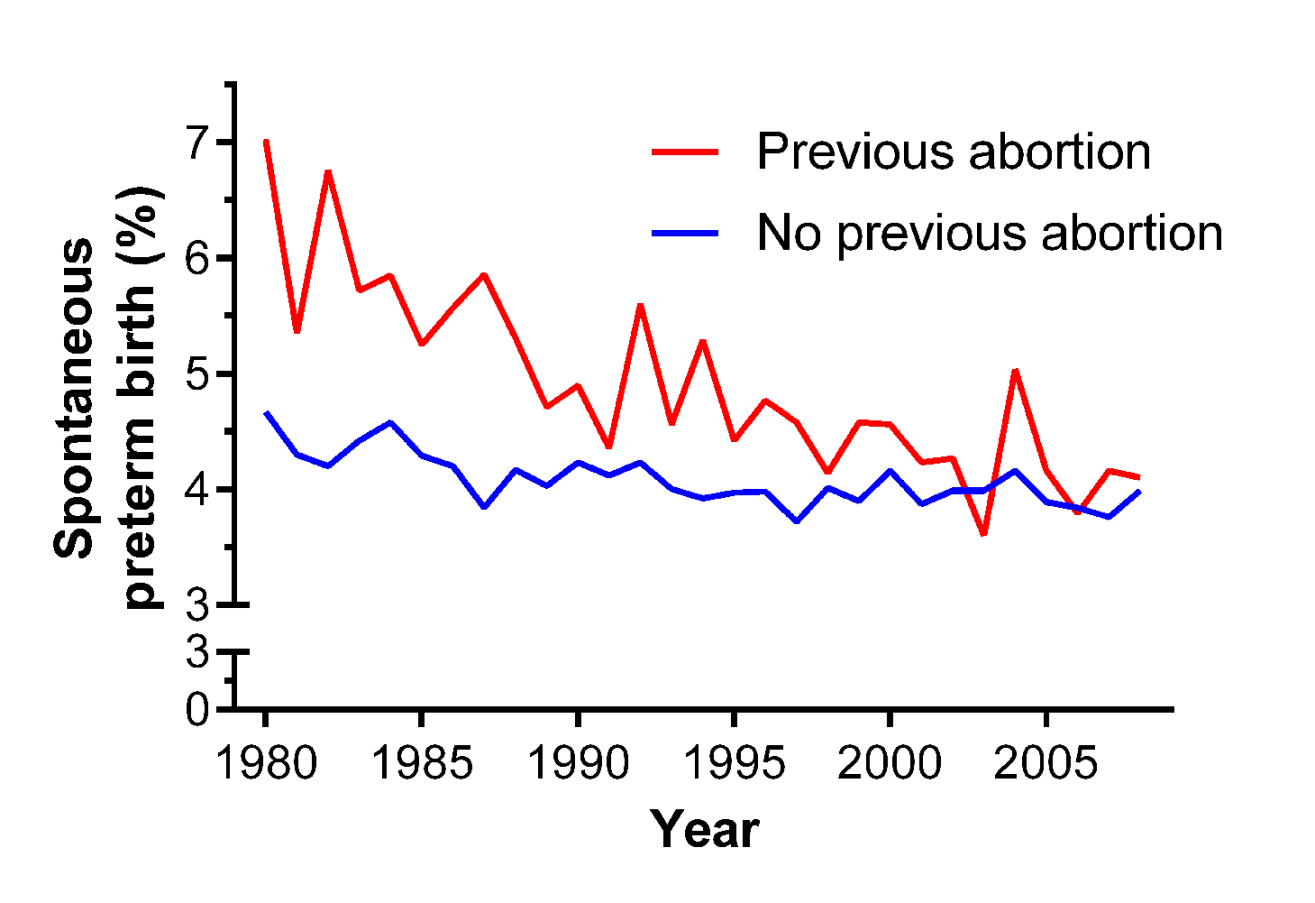
Crude rates of spontaneous preterm birth for nulliparous women with (*n* = 63,428) and without (*n* = 669,291) a past history of abortion in Scotland, 1980–2008.

The odds ratio for preterm birth associated with previous abortion significantly varied over the period 1980 to 2008 in both unadjusted (Figure 3A) and adjusted analyses (Figure 3B). There was a strong association between the risk of preterm birth and a one-unit increase in the number of previous abortions for births in 1980–1983 (adjusted odds ratio 1.32 [95% CI 1.21–1.43]), but this association progressively weakened with advancing year, and there was no association between previous abortion and the risk of preterm birth from 2000 onwards. When abortion was coded as any history of abortion versus no history, the interaction was virtually identical and was also highly statistically significant (data not shown). Plotting the rates of spontaneous preterm birth from 1980 to 2008, there was a substantial fall among nulliparous women with a history of abortion. However, the rates were essentially stable over the same period of time among nulliparous women with no history of abortion (Figure 4).

The univariate and multivariate analyses were repeated confined to births from 1992 to 2008, as smoking data were collected only over this period. The odds ratios (95% CI) for spontaneous preterm birth for one, two, and three or more previous abortions were, respectively, 1.17 (1.12–1.22), 1.48 (1.32–1.65), and 1.57 (1.18–2.08) in univariate analysis (*p* for trend <0.001), and 1.17 (1.13–1.22), 1.51 (1.35–1.68), and 1.64 (1.24–2.18) after adjusting for maternal characteristics including smoking (adjusted *p* for trend <0.001). Further, the interaction between previous abortion and year of birth for preterm birth was also minimally affected by adjustment for smoking status (Figure 3C). Maternal height was negatively associated with the risk of spontaneous preterm birth; however, the odds ratio for a 10-cm decrease in maternal height did not vary over the period 1980 to 2008 (Figure 3D).

**Figure 5:**
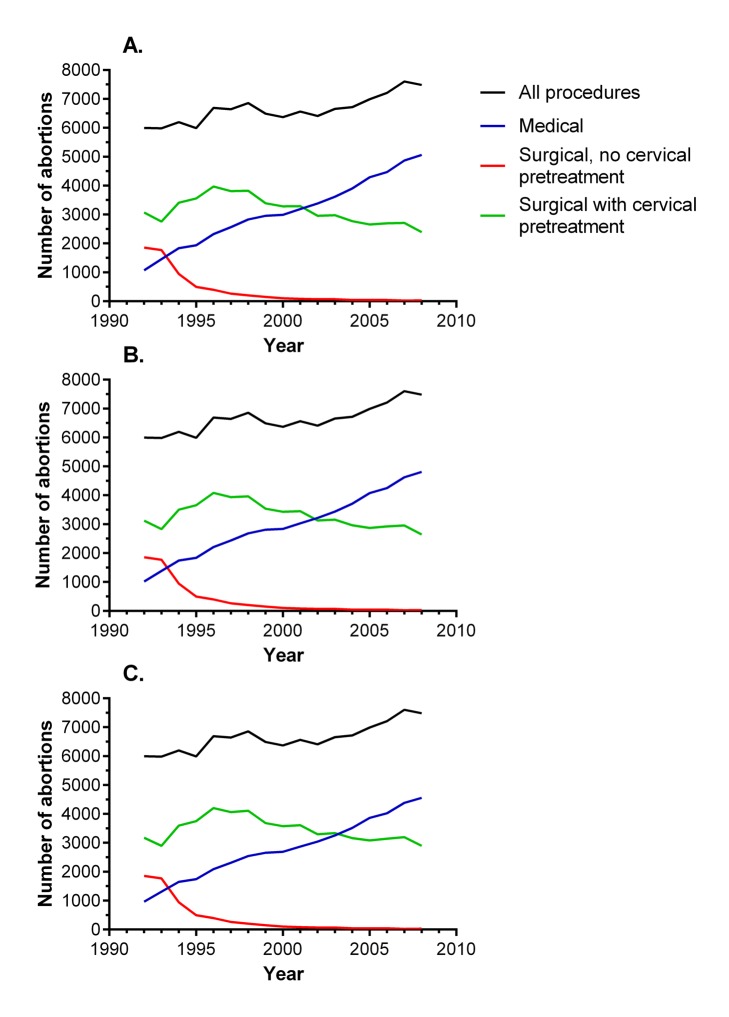
Annual numbers of abortions by method among nulliparous women in Scotland, 1992–2008. These data were aggregated and were not linked to SMR02 data. (A) Observed data. (B) Sensitivity analysis where 5% of medical procedures are re-classified as surgical procedures with cervical pre-treatment. (C) Sensitivity analysis where 10% of medical procedures are re-classified as surgical procedures with cervical pre-treatment.

Plotting the data on the methods of abortion used for nulliparous women in Scotland between 1992 and 2008 demonstrated that there was a dramatic rise in the number of abortions performed as medical procedures and a dramatic fall in the number of surgical procedures performed without pre-treatment of the cervix (Figure 5). When analysed as a proportion of the total annual abortions from 1992 to 2008, surgical abortions without use of cervical preparation decreased from 31% to 0.4%, and medical abortions increased from 18% to 68%. A proportion of medical abortions (generally less than 5% [3]) ultimately require a surgical procedure, but this information was not available for the sample. However, we performed sensitivity analyses of national trends in methods of abortion assuming that this affected 5% or 10% of all medical abortions, resulting in them being re-classified as surgical abortions with cervical preparation. The patterns of change observed between 1992 and 2008 were very similar with both assumptions (Figure 5).

## Discussion

We found a strong, independent relationship between a history of therapeutic abortion and the risk of first birth ending in spontaneous preterm delivery. For women with three or more previous abortions, there was an increased risk of neonatal death that was explained by the association with preterm delivery. However, the association between previous abortion and preterm birth was strongest in 1980–1983, progressively weakened over the next 15–20 y, and was no longer apparent from 2000 onwards. The change in the association was paralleled by increased use of medical termination of pregnancy, and pre-treatment of the cervix where the procedure was performed surgically. The most plausible explanation for these observations is that surgical termination of pregnancy without cervical pre-treatment is causally associated with the subsequent risk of spontaneous preterm birth and that declining use of this procedure led to the disappearance of the association between prior abortion and preterm birth from 2000 onwards. It is currently recommended that women considering abortion be informed of a possible increase in the risk of preterm birth in future pregnancies [3]. As there was no evidence of an independent association between abortion and future preterm birth in contemporary practice, the current recommendations about counselling women prior to abortion should be reconsidered.

The recommendation to counsel women about the future risk of preterm birth is based on a meta-analysis of 22 studies including 268,976 births [2]. Although the meta-analysis was published in 2009, the vast majority (>98%) of births included were prior to 2000. The meta-analysis did not explore the relationship between the study period and the strength of the association. Large-scale, population-based studies have been published since the meta-analysis. Bhattacharya et al. [11] reported an analysis of over 600,000 women giving birth in Scotland between 1981 and 2007 and found that women who had a single induced abortion were at increased risk of spontaneous preterm birth compared with women who had either had no previous pregnancies or had a previous live birth. In contrast, Klemetti et al. [12] analysed over 300,000 first births in Finland between 1996 and 2008 and found no association between one previous abortion and the overall risk of preterm birth. Neither study determined whether the association between previous abortion and preterm birth varied over the study period. Clearly, there are multiple possible explanations for the different findings in the two populations. However, one possible explanation, consistent with our own observations, is that the Finnish data covered a later period (1996–2008), whereas the previous analysis of Scottish data covered births between 1981 and 2007.

We speculate that changes in the methods used to achieve termination of pregnancy are the most plausible explanation for the loss of the association between previous abortion and the subsequent risk of preterm birth. However, previous studies have generated inconsistent results when analysing the risk of preterm birth in relation to the method of previous abortion. Analyses of approximately 12,000 Danish women of mixed parity [13] and approximately 8,000 nulliparous Finnish women [14] demonstrated no differences in outcome comparing women with previous medical versus surgical abortions. In contrast, the previous analysis of nationally collected data from Scotland discussed above demonstrated that previous surgical abortion was associated with spontaneous preterm first birth but that there was no independent association with previous medical abortion [11]. However, none of these studies sub-divided surgical abortions according to the use of cervical pre-treatment, and we have been unable to find any large-scale study that has done so. This information is not recorded in an electronic format by NHS National Services Scotland, and this question cannot, therefore, be directly addressed using the current data source.

Prostaglandins were introduced in the early 1980s to pre-treat (“prime”) the cervix prior to surgical abortion [15]. Because prostaglandins reduce the degree of mechanical dilation of the cervix required prior to a procedure, it is plausible that their use would decrease the risk of cervical damage and, hence, the risk of preterm birth in future pregnancies. In the aggregated data for all nulliparous women having abortions in Scotland (Figure 5), prostaglandins were used in about 60% of surgical procedures in 1992, and the proportion increased thereafter, such that surgical abortion without cervical pre-treatment had virtually disappeared by 2000. Hence, when considering changing methods of abortion over the period of study, there are two trends that emerged. The first was increasing use of cervical pre-treatment prior to surgical abortion, and the second was increasing use of non-surgical abortion. Detailed inspection of the data in Figures 3 and 5 sheds some light on the possible contribution of each of these to temporal changes in the association between previous abortion and preterm birth. The association had disappeared by 2000–2003. However, surgical abortion remained the more common method until 2001. If the risk of preterm birth associated with surgical abortion was unchanged over the study period, we would have expected to see an association between previous abortion and the risk of preterm birth among women delivering in 2000–2003, as the majority of these women who had a previous abortion would have had a surgical procedure. As there was no such association, we conclude that it is likely that the risk of preterm birth following surgical abortion declined over the period of study, and this is most plausibly explained by increasing use of cervical pre-treatment. Consistent with this interpretation, the Danish and Finnish studies that observed no difference between medical and surgical abortions analysed procedures performed during the periods 1999–2004 and 2000–2009, respectively. In contrast, the analysis of Scottish data that found higher risks of preterm birth following surgical abortions compared with medical abortions included procedures performed between 1981 and 2007. Hence, the existing literature supports our interpretation, but, clearly, studies directly addressing the effect of cervical pre-treatment on the risk of preterm birth following a surgical abortion should be a priority for future research.

The above interpretation assumes a causal association between surgical abortion without cervical pre-treatment and preterm birth, and there are a number of aspects of the current analysis that are supportive of a causal relation. First, there was evidence of a dose–response relationship: the risk of a preterm first birth increased with the number of previous abortions. Second, when analysed by the type of preterm birth, we observed that previous abortion was independently associated with spontaneous preterm birth, but not induced preterm birth. The specificity is again supportive of a causal association. Third, adjustment for maternal characteristics was without material effect on the nature or strength of the association. Fourth, the loss of the relationship between previous abortion and preterm birth over the period of study indicates that whatever mechanism linked the two, causal or confounding, the factor disappeared over the period 1980 to 2000. The fact that the specific procedure most strongly associated with cervical damage (surgical abortion without cervical pre-treatment [4]) essentially disappeared over the same period of time indicates a biologically plausible mechanism. Moreover, there is no obvious confounding factor, to our knowledge, that changed so dramatically over the same period of time. Finally, the interaction between year of birth and previous abortion was also specific: there was no comparable interaction between year of birth and short stature.

The World Health Organization has recently reviewed the global problem of preterm birth, and addressing it has been described by the Secretary-General of the United Nations as a “crucial aspect” of the work required to achieve the 2015 Millennium Development Goals [16]. It is estimated that, globally, 15 million babies are born preterm, and over 1 million infants die because of preterm birth every year. The World Health Organization report identified many potential strategies to reduce the burden of morbidity and mortality of preterm birth. A recent study evaluated the impact of five of these and found limited potential for reduction in the global numbers of preterm births [17]. However, neither report addressed the possible effects of methods used to achieve termination of pregnancy. In 2008, there were more than 40 million terminations of pregnancy performed worldwide [1], with approximately 6 million procedures taking place in high-income countries and the majority taking place in low-and middle-income countries. Many women having an abortion will subsequently have an ongoing pregnancy in later life. The current analysis suggests that modernising methods of abortion within a region where purely surgical methods are widespread may significantly reduce the subsequent burden of morbidity and mortality related to preterm birth. The widespread use of abortion and the global problem of preterm birth are such that understanding the relationship between methods of abortion, surgical and medical, and the risk of preterm birth needs to be improved.

The current study has a number of limitations. Because we used routinely collected data, the amount of information on each woman was more limited than would have been available in a prospective study, such as ethnicity and whether access to abortion was equitable across groups. However, NHS National Services Scotland has offered free abortion services to all residents of Scotland since 1967 under the auspices of the Abortion Act 1967. The data on secular trends in abortion did not distinguish between successful medical abortions and those where surgical evacuation of the uterus was ultimately required. However, failed medical abortions generally involve treatment with both anti-progestogens and prostaglandins, which would be expected to soften and dilate the cervix. We speculate that surgical abortions with prior cervical pre-treatment may be similarly protective as medical abortions against the risk of future preterm birth. Hence, we would anticipate that medical abortion would be associated with a low risk of future preterm birth whether a surgical procedure was ultimately required or not. The current analysis reports only the associations between prior abortion and the outcome of the first birth. Hence, it is not clear whether these conclusions would be generalisable to parous women. We considered including second and subsequent births. However, this would have complicated the interpretation of the data as women might use abortion selectively on the basis of the outcome of prior births. Finally, although the interaction remained statistically significant after adjustment for maternal characteristics, we cannot exclude the possibility that the change occurred through the effect of an unmeasured confounder, or due to some other changes in abortion practice such as the gestational age at which procedures are performed. This might be addressed by determining whether a similar interaction is observed in other countries that have introduced modern methods of abortion over a period of complete national data collection. It is also possible that other national datasets might include information on the method of previous abortion, including use of cervical pre-treatment: such studies could be used to test our interpretation directly. It is not, however, plausible that the changes observed were due to improved care of women at risk of preterm birth, as there are few effective interventions to reduce the risk of this outcome in nulliparous women [18].

In conclusion, we have shown that previous abortion was a risk factor for preterm birth among nulliparous women in Scotland prior to 2000. However, increased use of medical methods of abortion and of cervical pre-treatment prior to surgical abortion has been paralleled by a disappearance in the association. We believe that it is plausible that modernising methods of termination of pregnancy worldwide may be an effective long-term strategy to reduce future rates of preterm birth.

## Supporting Information

Table S1 **Multivariate logistic regression analysis of the association between neonatal death and previous abortion (coded as categories).**
(DOCX)

Table S2 **Multivariate logistic regression analysis of the association between neonatal death and previous abortion (coded as binary variable).**
(DOCX)

Table S3 **Multivariate logistic regression analysis of the association between neonatal death and previous abortion (coded as continuous variable).**
(DOCX)

## Abbreviations

NHS: National Health Service
SMR02: Scottish Morbidity Record 02
SSBIDS: Scottish Stillbirth and Infant Death Survey
