# Supplementary material for: Changes in Association between Previous Therapeutic Abortion and Preterm Birth in Scotland, 1980 to 2008: A Historical Cohort Study
Source: PLoS Med. 2013 Jul 9;10(7):e1001481. doi: 10.1371/journal.pmed.1001481 (PMC3706322; doi:10.1371/journal.pmed.1001481)
Supplement: Table S1 — Multivariate logistic regression analysis of the association between neonatal death and previous abortion (coded as categories). (DOCX) [file pmed.1001481.s001.docx]

**Supplementary Table 1: Multivariate logistic regression analysis of the association between neonatal death and previous abortions (coded as categories)**

|  | Odds Ratio (95%CI) | | | | | | |
| --- | --- | --- | --- | --- | --- | --- | --- |
|  | All Preterm Birth | Spontaneous Preterm Birth | Induced Preterm Birth | Preterm Birth (24-28 weeks) | Preterm Birth (29-32 weeks) | Preterm Birth (33-36 weeks) | Neonatal Death* |
| History of Therapeutic Abortion |  |  |  |  |  |  |  |
| 0 Abortions | 1.00 | 1.00 | 1.00 | 1.00 | 1.00 | 1.00 | 1.00 |
|  | ref | ref | ref | ref | ref | ref | ref |
| 1 Abortion | 1.11 | 1.17 | 1.00 | 1.05 | 1.06 | 1.13 | 1.07 |
|  | (1.07-1.15) | (1.12-1.22) | (0.94-1.06) | (0.92-1.20) | (0.97-1.16) | (1.08-1.17) | (0.88-1.31) |
| 2 Abortions | 1.32 | 1.51 | 1.05 | 2.06 | 1.25 | 1.28 | 1.14 |
|  | (1.20-1.45) | (1.35-1.68) | (0.86-1.21) | (1.54-2.74) | (0.99-1.58) | (1.15-1.42) | (0.64-2.01) |
| 3+ Abortions | 1.45 | 1.64 | 1.17 | 3.33 | 1.46 | 1.30 | 2.68 |
|  | (1.15-1.84) | (1.24-2.18) | (0.78-1.74) | (1.83-6.06) | (0.82-2.59) | (0.98-1.72) | (1.00-7.20) |
| History of Miscarriage |  |  |  |  |  |  |  |
| No History of Miscarriage | 1.00 | 1.00 | 1.00 | 1.00 | 1.00 | 1.00 | 1.00 |
|  | ref | ref | ref | ref | ref | ref | ref |
| 1+ Miscarriages | 1.25 | 1.19 | 1.37 | 1.72 | 1.36 | 1.20 | 1.40 |
|  | (1.22-1.29) | (1.15-1.23) | (1.31-1.43) | (1.57-1.90) | (1.28-1.46) | (1.16-1.24) | (1.19-1.63) |
| Height  (per cm) | 0.97 | 0.98 | 0.97 | 0.97 | 0.97 | 0.97 | 0.99 |
|  | (0.97-0.98) | (0.98-0.98) | (0.96-0.97) | (0.96-0.97) | (0.97-0.97) | (0.97-0.98) | (0.98-1.00) |
| Marital Status |  |  |  |  |  |  |  |
| Married | 1.00 | 1.00 | 1.00 | 1.00 | 1.00 | 1.00 | 1.00 |
|  | ref | ref | ref | ref | ref | ref | ref |
| Non-Married | 1.22 | 1.27 | 1.10 | 1.31 | 1.30 | 1.19 | 1.14 |
|  | (1.19-1.25) | (1.23-1.30) | (1.05-1.16) | (1.19-1.45) | (1.22-1.38) | (1.16-1.23) | (0.99-1.32) |
| Maternal Age  (per year) | 1.01 | 0.99 | 1.04 | 1.00 | 1.01 | 1.01 | 1.00 |
|  | (1.00-1.01) | (0.99-0.99) | (1.04-1.04) | (0.99-1.00) | (1.00-1.01) | (1.00-1.01) | (0.99-1.01) |
| Deprivation Category |  |  |  |  |  |  |  |
| 1 | 1.00 | 1.00 | 1.00 | 1.00 | 1.00 | 1.00 | 1.00 |
|  | ref | ref | ref | ref | ref | ref | ref |
| 2 | 1.03 | 1.03 | 1.04 | 1.20 | 1.06 | 1.01 | 1.24 |
|  | (0.98-1.09) | (0.96-1.09) | (0.95-1.13) | (0.96-1.50) | (0.93-1.21) | (0.96-1.07) | (0.89-1.72) |
| 3 | 1.06 | 1.04 | 1.10 | 1.23 | 1.07 | 1.05 | 1.26 |
|  | (1.01-1.12) | (0.98-1.11) | (1.01-1.19) | (1.00-1.52) | (0.94-1.21) | (0.99-1.11) | (0.91-1.73) |
| 4 | 1.07 | 1.05 | 1.12 | 1.33 | 1.10 | 1.05 | 1.33 |
|  | (1.02-1.13) | (0.98-1.11) | (1.04-1.22) | (1.08-1.64) | (0.97-1.24) | (1.00-1.11) | (0.97-1.82) |
| 5 | 1.13 | 1.08 | 1.21 | 1.44 | 1.16 | 1.10 | 1.38 |
|  | (1.07-1.19) | (1.02-1.15) | (1.11-1.32) | (1.16-1.79) | (1.02-1.32) | (1.04-1.17) | (1.00-1.92) |
| 6 | 1.08 | 1.05 | 1.14 | 1.22 | 1.10 | 1.06 | 1.35 |
|  | (1.02-1.14) | (0.98-1.12) | (1.04-1.24) | (0.98-1.53) | (0.97-1.26) | (1.00-1.13) | (0.97-1.90) |
| 7 | 1.18 | 1.17 | 1.18 | 1.49 | 1.22 | 1.16 | 1.31 |
|  | (1.12-1.25) | (1.09-1.25) | (1.08-1.30) | (1.18-1.88) | (1.06-1.41) | (1.09-1.23) | (0.91-1.87) |
| Year of Delivery  (per year) | 1.00 | 0.99 | 1.02 | 1.00 | 1.00 | 1.00 | 0.97 |
|  | (1.00-1.00) | (0.99-1.00) | (1.01-1.02) | (1.00-1.01) | (0.99-1.00) | (1.00-1.00) | (0.96-0.98) |

*CI denotes confidence interval*

** Neonatal death data was only available from 1985 onwards*
